# An analysis on the effect of body tissues and surgical tools on workflow recognition in first person surgical videos

**DOI:** 10.1007/s11548-024-03074-6

**Published:** 2024-02-27

**Authors:** Hisako Tomita, Naoto Ienaga, Hiroki Kajita, Tetsu Hayashida, Maki Sugimoto

**Affiliations:** 1 Graduate School of Science and Technology, Keio University, Yokohama, 2238522 Japan; 2 Institute of Systems and Information Engineering, University of Tsukuba, Tsukuba, 3058573 Japan; 3 Department of Plastic and Reconstructive Surgery, Keio University School of Medicine, Tokyo, 1608582 Japan; 4 Department of Surgery, Keio University School of Medicine, Tokyo, 1608582 Japan

**Keywords:** The first-person-view surgical video, Body tissues, Surgical tools, Segmentation, Workflow recognition

## Abstract

****Purpose**:**

Analysis of operative fields is expected to aid in estimating procedural workflow and evaluating surgeons’ procedural skills by considering the temporal transitions during the progression of the surgery. This study aims to propose an automatic recognition system for the procedural workflow by employing machine learning techniques to identify and distinguish elements in the operative field, including body tissues such as fat, muscle, and dermis, along with surgical tools.

****Methods**:**

We conducted annotations on approximately 908 first-person-view images of breast surgery to facilitate segmentation. The annotated images were used to train a pixel-level classifier based on Mask R-CNN. To assess the impact on procedural workflow recognition, we annotated an additional 43,007 images. The network, structured on the Transformer architecture, was then trained with surgical images incorporating masks for body tissues and surgical tools.

****Results**:**

The instance segmentation of each body tissue in the segmentation phase provided insights into the trend of area transitions for each tissue. Simultaneously, the spatial features of the surgical tools were effectively captured. In regard to the accuracy of procedural workflow recognition, accounting for body tissues led to an average improvement of 3 % over the baseline. Furthermore, the inclusion of surgical tools yielded an additional increase in accuracy by 4 % compared to the baseline.

****Conclusion**:**

In this study, we revealed the contribution of the temporal transition of the body tissues and surgical tools spatial features to recognize procedural workflow in first-person-view surgical videos. Body tissues, especially in open surgery, can be a crucial element. This study suggests that further improvements can be achieved by accurately identifying surgical tools specific to each procedural workflow step.

## Introduction

Surgical videos serve as educational resources for learning operative techniques and procedures. Conventionally, student doctors and residents have had opportunities to learn operative skills by observing open surgery. However, this traditional approach presents a significant challenge: surgeons and their assistants often obstruct the view of the operative field. This obstruction makes it challenging for observers to consistently witness crucial manipulations. To address the issue, capturing videos of the operative field is beneficial.

In open surgery, first-person-view videos are often used to provide an unobstructed view of the operative field. The surgeon’s movements during surgery can be recorded and played back to facilitate learning after open surgery. However, recording and watching first-person-view surgical videos pose challenges. Firstly, the technology for recording open surgery video is limited and more difficult than endoscopic surgery. To record first-person-view videos, surgeons typically wear cameras [1]. Saun et al. [2] have researched various technologies available for this purpose. Secondly, a persistent issue in surgical videos is their length. To effectively learn operative skills, it is necessary to skip non-essential scenes, such as gloves being exchanged, and summarize the videos. Manual video editing is very time-consuming, often exceeding the actual video duration [3]. Automated video editing can help medical professionals save time. Procedural workflow recognition is one of the most effective methods to summarize the videos because it allows us to estimate the progress of the surgery and facilitate segmentation of essential and non-essential scenes.

The conditions of the operative field vary across different workflows and evolve over time. Recognizing different elements of the surgical scene becomes possible by leveraging changes in and body tissues and surgical tools. Khalid et al. [4] detected the surgical tool tips to measure surgical performance and Quadir et al. [5] utilized Mask R-CNN [6] to detect polyps within internal body tissues. Irshad et al. [7] detected collaborative scenes from surgeons’ multiple hands and gaze locations. Yoshida et al. [8] demonstrated the identification of critical areas in first-person-view surgical videos using surgeons’ hands and gaze points. Their contribution involves classifying these scenes as either essential or non-essential. Their approach utilized region mask images of body tissues or surgical tools to recognize operative field situations. While bounding boxes are effective [9] for operative field detection, incorporating more detailed spatial information, such as mask images, can recognize more details of the operative field.

Various studies have been conducted on methods and datasets for recognizing procedural workflow. Nakawala et al. used image features convoluted by CNN and input to LSTM [10] in their Deep-Onto [11]. Their dataset was Nephrec9 [12], which contains nine surgical videos recorded during a robot-assisted partial nephrectomy. The process transitions were estimated using discrete-time Markov chain through conditional branching. Pan et al. used Swin Transformer [13] which had image attention mechanisms to estimate the surgical process. They used Cholec80 dataset [14], which includes 80 cholecystectomy surgeries performed by 13 surgeons. These studies primarily focused on endoscopic surgery.

In this paper, we collect first-person-view open surgery videos and detect body tissues and surgical tools regions. The precision of procedural workflow recognition is evaluated by comparing models using detected body tissues and surgical tools regions with a baseline network model. Our research questions are below:

**RQ1** Is the tracking of body tissues area a valid factor in the estimation of the procedural workflow?

**RQ2** To what extent can the accuracy of procedural workflow recognition be improved by machine learning that takes into account information on body tissues and surgical tools?

**Fig. 1:**
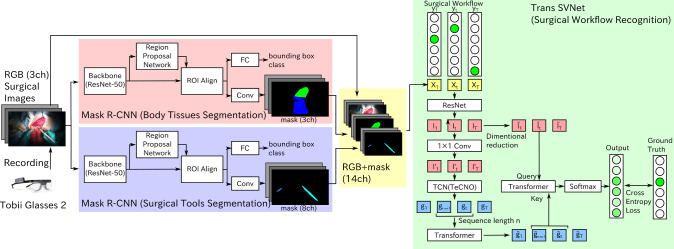
First-person-view surgical video recorded by a wearable camera was downsampled and passed through Mask R-CNN to identify body tissues and surgical tools. RGB surgical images and region masks were concatenated by channel direction to create multi-channel data. The procedural workflow was recognized after passing through Trans-SVNet

This study investigates whether the accuracy in procedural workflow recognition of first-person-view breast surgery videos improves with machine learning that explicitly identifies internal body tissues. Figure 1 shows our system overview.

## Dataset

### Video data collection

We used surgical videos recorded in breast cancer surgery by wearable cameras, Tobii Glasses2 and Tobii Glasses3 [15]. Tobii Glasses were designed as a pair of eyeglasses equipped with a scene camera and eye tracking sensors. The scene camera recorded first-person-view videos at a rate of 25 frames per second (fps). We collected nine first-person-view videos from the viewpoint of the surgeon. The duration of each video ranged between one and two hours. We attempted to record the surgery from the beginning to the end.

The full dataset used in this study was filmed after obtaining written informed consent, based on a protocol approved by the Ethics Committee of the Keio University School of Medicine (20180026).

### Segmentation data

In breast cancer surgery, distinct surgical tools were used for each workflow and three body tissues were observed (dermis, fat, and muscle). To train a machine learning model to detect body tissues and surgical tools, we annotated 400 images for body tissue segmentation and, 508 images for surgical tools segmentation. We used VGG Image Annotator [16] to label all regions. The regions were represented as polygons created by connecting dots outlining the contours. If the region of a single item was divided by an obstacle such as a hand or another item, each region was labeled as a separate region. Table 1 indicates the number of annotated images and instances. We divided the dataset for train, validation and test. In the case of body tissues, the ratio of *train* : *validation* : *test* was 14 : 6 : 1, while for surgical tools, the rate was 8 : 2 : 1. The dataset were split randomly.

**Table 1:** Number of labeled images for body tissues and surgical tools segmentation. The split rate for body tissues segmentation, $train:validation:test=14:6:1$ and for surgical tools segmentation, the rate was 8 : 2 : 1

	Train+validation+test	
Regions	Images	Instances
Dermis	61	85
Fat	391	638
Muscle	216	274
Pen	26	33
Syringe	57	98
Scalpel	44	65
Electrical scalpel	144	160
Hook	114	204
Tweezers	161	373
Forceps	54	98
Needle holders	59	71

### Workflow recognition data

We labeled the videos as six class labels using ELAN [17] annotation tool. The classification was instructed by a breast surgeon. The workflow classes were Before Incision (BI), Making Flap (F), Mammary Gland Dissection and Lymph Node (ML), Irrigation and Drain Insertion (ID), Skin Suture (SS) and Other Behavior (OB). Most surgeries proceeded in the aforementioned order. The OB class included the scene where the surgeon did not look at the operative field. However, if the surgeon glanced out of the operative field and then immediately returned to the operative field, the scene was not included in the OB class. The BI class included marking with a pen and Bosmin injection. Table 2 indicates the number of images used for training, validating, and testing each workflow. It is important to note that the datasets for segmentation and workflow recognition were distinct and separate entities.

**Table 2:** Number of labeled images for procedural workflow recognition

Workflow	The number of images		
	Train	Validation	Test
Before Incision	834	143	180
Making Flap	5213	2037	1352
Mammary Gland Dissection and Lymph Node	10,142	1757	2620
Irrigation and Drain Insertion	1633	303	456
Skin Suture	4211	801	724
Other Behavior	6506	1453	2642
Total	28,539	6494	7974

## Body tissues and surgical tools segmentation

### Network and image augmentation

We used Mask R-CNN [6] as our framework for body tissues and surgical tools segmentation. In the training of surgical tools detection, we utilized pre-training models to obtain features closely related to the domain of surgical tools. We compared three pre-trained models with datasets: ImageNet [18], EndoVis2017 dataset [19] and both of them. For EndoVis2017, we trained the Mask R-CNN model with the dataset for 35 epochs from scratch. For ImageNet, we employed the Pytorch pre-trained parameter for the Mask R-CNN model with the dataset. For ImageNet + EndoVis2017, initially, we loaded the PyTorch pre-trained parameter with ImageNet, then the model was trained with EndoVis2017 to obtain surgical tool’s tips features for 44 epochs. After that, all three pre-trained models were fine-tuned with our dataset. For the assessment of the pre-training and fine-tuning models, each model was trained five times for fivefold cross-validation of training and validation datasets, and the evaluation scores were obtained as the average of each five testing.

On the other hand, in body tissues training, we did not use any pre-trained weights, because the accuracy of segmentation was worse when we applied a pre-trained model. One of the reasons for worse was considered that the images used for pre-train were different from the surgical images.

As for other parameters, we used Adam optimizer and learning rate was $1.0 \times 10^{-4}$. In training, data augmentations were applied, such as horizontal flip, random crop, brightness changing, and Gaussian blurring. Each image processing technique was applied with a 50 % probability. The kernel size of the Gaussian filter was $5 \times 5$. To adjust the brightness, we randomly selected a value for $\alpha$ between 0.5 and 1.5, and a value for $\beta$ between -50 and 50. Any pixel values that fell below 0 or above 255 were rounded to 0 or 255, respectively. In first-person-view surgical videos, changes in brightness and motion blur occurred suddenly. These augmentations contributed to detecting regions even when the camera images were unclear.

### Result

**Table 3:** IoU and AP of body tissues segmentation

Regions	IoU(%)	AP(%)
Dermis	29.22	56.19
Fat	**67**.**29**	75.51
Muscle	52.32	**89**.**11**
Mean of total	49.61	73.60

The highest IoU or AP value for each region is shown in bold

**Table 4:** IoU and AP of surgical tools segmentation

	Pre-trained dataset					
Regions	EndoVis2017		ImageNet		ImageNe+EndoVis2017	
	IoU(%)	AP(%)	IoU(%)	AP(%)	IoU(%)	AP(%)
Pen	3.15	24.14	34.51	**77**.**05**	**38**.**06**	75.89
Syringe	0.14	4.18	**21**.**62**	**73**.**07**	18.41	68.27
Scalpel	3.10	31.79	18.77	68.08	**20**.**45**	**73**.**17**
Electrical scalpel	20.59	75.92	29.18	79.34	**32**.**69**	**88**.**78**
Hook	2.68	55.27	3.61	52.01	**4**.**42**	**56**.**04**
Tweezers	2.00	29.84	7.14	**59**.**61**	**8**.**36**	54.35
Forceps	0.40	22.92	2.88	33.42	**3**.**87**	**41**.**14**
Needle holders	3.37	31.72	12.66	33.42	**19**.**01**	**55**.**17**
Mean of total	4.42	34.47	16.30	61.72	**18**.**15**	**64**.**10**

The method that estimates each region with the highest IoU or AP value is highlighted in bold

**Fig. 2:**
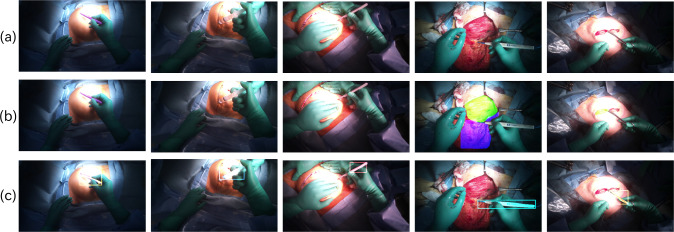
Example of the results of segmentation. **a** RGB surgical images. **b** The result of body tissues segmentation. Fat is painted magenta and muscle is painted green. Dermis is also predicted in the center image, but the region size is small. **c** The result of surgical tools segmentation. Different tools are painted in different colors with bounding boxes

IoU and mean Average Precision (mAP) were calculated to evaluate the segmentation accuracy with a test dataset independent of the training dataset. Table 3 and Table 4 indicate each result of segmentation. In body tissues, fat and muscle are higher than dermis because their regions are larger than dermis and they have a large number of dataset. For surgical tools, thinner tools and tools with similar shapes to others have lower detection accuracy. However, by utilizing ImageNet + EndoVis2017 for pre-training, the overall IoU and mAP metrics surpassed the individual pre-training models. Figure 2 shows snapshots of the body tissues and tool segmentation.

The top row indicates input surgical images. From left to right: marking with pen, bosmin injection, making flap, mammary gland dissection, and skin suture. The remaining rows illustrate the output of segmentation.

### Discussion

In body tissues segmentation, accuracy was significantly influenced by data imbalance. Fat and muscle were seen in most of the scenes, and their region sizes were larger than dermis. In comparison with that dermis was only seen the scene of beginning to open the breast. When we attempted to evenly collect training images from each scene, the number of body tissues included in surgical images became imbalanced. Consequently, the accuracy of fat and muscle detection was higher than dermis.

Identifying surgical tools can be challenging due to their thin regions and small number of pixels. Additionally, some tools, such as forceps and needle-holders, can be similar in appearance, leading to low identification accuracy. Brightness and reflection are also factors of low detection accuracy. In particular, metallic luster surgical tools sometimes showed blown out highlights areas and unclear boundaries under the light. The utilization of ImageNet as the pre-trained dataset resulted in notably lower accuracy for certain surgical tools, such as hook, forceps, tweezers, and needle-holders, in comparison with pen, syringe, scalpel, and electrical-scalpel. This discrepancy is attributed to the fact that ImageNet primarily consists of general objects, and the plastic components of surgical tools exhibit features that are more analogous to those present in the pre-trained data. Forceps are less frequently used compared to other surgical tools, and instances of forceps overlapping with each other are common. Therefore, under all of situations, it is presumed that the accuracy of instance segmentation is lower compared to other surgical tools.

## Workflow recognition

### Workflow classification network

The network of workflow classification was Trans-SVNet [20]. In Trans-SVNet, ResNet [21] was trained for spatial embedding, and temporal convolutional network(TCN) was trained for temporal embedding. A sequence of temporal embedding was fed into one of the Transformer layers. Then, another Transformer was trained to use these two embeddings as spatial-temporal hybrid embedding. The network’s output consisted of the probability of each workflow in the range [0, 1], determined through the application of softmax, and the backward propagation of the cross-entropy loss. In this study, three different inputs were compared to verify identification accuracy. The first input consisted solely of RGB images, which is baseline(RGB). The second input consisted RGB images with three-channel body tissue masks (RGB+BT). The third input consisted RGB images with three-channel body tissues and eight-channel surgical tools masks (RGB+BT+ST).

Seven complete surgical videos were used in the training. Five of them were used for the training model, one was used for validation, and the remaining videos were used for testing. All surgical videos were recorded at 25 fps and were downsampled to 1 fps. For temporal embedding, the sequence size was set to 30 s. In Trans-SVNet, data augmentations were applied, including horizontal flipping, random cropping, random rotation, and color adjustments. When the input was RGB surgical images and region masks, color jitter was applied only RGB surgical images not region masks, and other data augmentations were applied to both types of data.

### Result

**Fig. 3:**
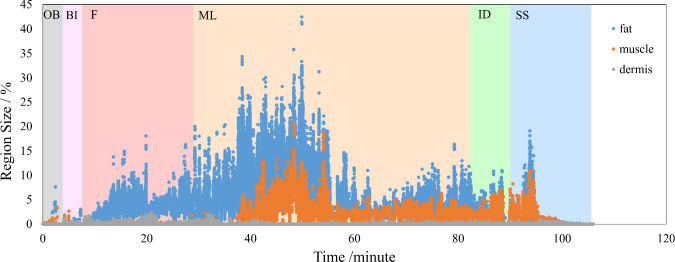
Graph illustrates the changes in the sizes of body tissue regions and the workflows. The abbreviations used in the graph are as follows: OB (other behavior), BI (before incision), F (making flap), ML (mammary gland dissection and sentinel lymph node biopsy), ID (irrigation and drain insertion), and SS (skin suture)

Figure 3 indicates an example of the transition of body tissues region sizes. The region sizes of body tissues were expressed as percentages relative to the full image pixels. In the first half of the surgery, there were mainly fat and dermis regions, and muscle regions appeared in the midfield. As the incision progressed, the region size of fat and muscle increased. During the Skin Suture phase, the size of these regions rapidly decreased. When the surgeon did not focus on the operative field, the values were 0. The observed transition pattern in the sizes of body tissues in breast surgery videos appears to be consistent across the recorded videos.

**Fig. 4:**
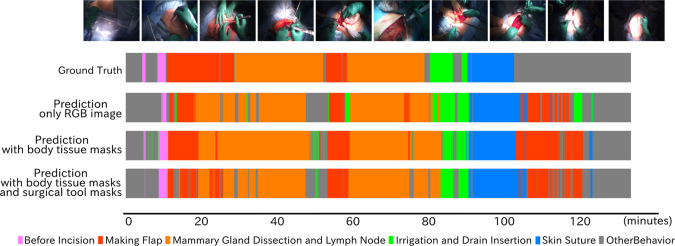
Four ribbon graphs show the temporal evolution of the procedural workflow. The top images show a snapshot of the operative field at regular intervals

Table 5 shows the accuracy of workflow recognition using Trans-SVNet. Precision, recall, and F1 were calculated for each workflow. The higher value in each workflow is highlighted in bold. Overall, the addition of body tissues and surgical tools masks resulted in higher precision and recall compared to using only RGB images. Specifically, the RGB+BT method achieved nearly 3 % higher precision than the baseline, while the RGB+BT+ST method achieved 5 % higher precision. Additionally, both region-aware methods achieved 4 % higher recall than the baseline method. Similarly for F1, the result was improved by 3 % when the input was RGB+BT, and RGB+BT+ST was nearly 5 % higher than the baseline. Figure 4 compares the predicted workflow among the three different inputs. Although each input has some short-term incorrect predictions, the figure indicates RGB+BT showed fewer errors than other models.

**Table 5:** Each workflow’s comparison of precision, recall and F1 by input type

	Input	BI	F	ML	ID	SS	OB	Total
Precision(%)	Baseline(RGB)	**83.45**	47.59	73.44	48.76	76.91	68.71	66.49
	RGB+BT	77.45	42.67	73.91	53.41	**85.38**	**82.76**	69.27
	RGB+BT+ST	79.43	**51.92**	**77.32**	**73.90**	77.27	70.32	**71.69**
Recall(%)	Baseline(RGB)	36.67	42.38	78.93	**81.80**	94.75	**57.68**	65.37
	RGB+BT	**74.44**	**59.24**	**87.25**	48.02	**95.99**	50.34	69.22
	RGB+BT+ST	62.22	56.95	81.83	62.72	94.89	**61.24**	**69.98**
F1(%)	Baseline(RGB)	50.97	44.83	76.09	61.10	84.90	**62.71**	65.93
	RGB+BT	**75.92**	49.61	**80.03**	49.26	**90.37**	62.60	69.24
	RGB+BT+ST	69.78	**54.32**	79.51	**67.85**	85.18	**65.47**	**70.82**

The three input patterns with the highest precision, recall, and F1 values are bolded for each workflow

Between 15 and 30 min, all prediction models misidentified the Making Flap as the Mammary Gland Dissection and Lymph Node due to their similar appearance and the intermittent nature of these workflows. In Making Flap phase, there were a large number of prediction errors, resulting in lower precision and recall. Furthermore, during the Other Behavior phase between 100 min and 120 min, it was mistakenly identified as the Making Flap and other workflows. In this scene, the surgeons held around the sutured skin with their hands, and it was similar to the scene at the beginning of the skin incision. At the last 20 min of the surgery, the models of RGB+BT and RGB+BT+ST estimated the workflow correctly despite of errors in the body tissue and surgical tool segmentation.

### Discussion

Figure 4 indicates that the short-time errors in the RGB model were reduced in the RGB+BT and RGB+BT+ST models by considering the transition of body tissues. For RQ1, we found that tracking body tissues region was indeed a valid factor to improve the accuracy of procedural workflow recognition. For RQ2, the result suggests that both body tissues and surgical tools contribute to the improvement of procedural workflow recognition accuracy. We revealed that explicitly representing the state of the operative field leads to a reduction in ambiguity and improved precision in procedural workflow recognition. Nevertheless, challenges persist in achieving accurate segmentation. However, there is potential for future improvements in accuracy through advancements in the methodology.

This study has demonstrated the potential for further development in video-based surgical education for open surgery. Jason L. Green et al. [22] revealed that video-based education, supplementing educational tools such as texts, demonstrated statistically significant increases in anatomical and procedural knowledge, as well as improvement in surgical performance. The findings in this study contributes to making educational video contents by extracting relevant video clipping and creating chapters in consideration of body tissues and surgical tools.

## Limitations

In this study, workflow classes were limited to a specific resolution. It is possible to consider more detailed classes. For instance, workflows can be divided based on differences in the surgical tools used between Irrigation and Drain Insertion. In future work, it is expected to achieve a more detailed class classification using operative field information.

The identification accuracy of the surgical tools was lower than we had expected. Given the inherent significance of surgical tools in the surgical process, their effective identification is crucial for workflow recognition. We expect that achieving high discrimination accuracy in segmentation could lead to a more robust workflow recognition.

Although this study was limited to breast surgery, it is possible to recognize workflows by applying observed body tissues and surgical tools used in other surgeries. However, given the variations in features of internal body tissues and surgical tools across different surgeries, there is a need to create dedicated datasets. It is anticipated that future developments may lead to the creation of a general-purpose dataset to address these challenges.

## Conclusion

In this study, we investigated the impact of the temporal transition of the body tissues and surgical tools to recognize procedural workflow in first-person-view surgical videos. Our experimentation revealed that employing classifiers for both body tissues and surgical tools enhanced the accuracy of procedural workflow recognition in breast surgery videos. Both body tissues and surgical tools were contributed to the recognition accuracy as spatial-temporal features of the operative field.
